# Recovery from borderline personality disorder in relation to the concept of “a life worth living”: a therapist perspective

**DOI:** 10.1186/s40479-026-00352-4

**Published:** 2026-09-04

**Authors:** Josephine Ljungberg

**Affiliations:** Department of Psychology, Stockholm University, Stockholm, SE-114 19 Sweden

**Keywords:** Borderline Personality Disorder (BPD), Dialectical Behavior Therapy (DBT), Recovery, A life worth living

## Abstract

**Background:**

Despite increased interest in clients’ subjective experiences of recovery from borderline personality disorder (BPD), qualitative research exploring how Dialectical Behavior Therapy (DBT) therapists conceptualize recovery and its relationship to DBT’s overarching treatment goal, “a life worth living,” remains scarce. The aim of the present study was to explore DBT therapists’ descriptions of recovery sufficient for treatment termination in clients with BPD, as well as to examine its relation to the concept of a life worth living.

**Methods:**

Eight semi-structured interviews with DBT therapists were conducted and analyzed using reflexive thematic analysis.

**Results:**

Therapists described recovery as a transition from suffering to a life of normal freedom, viewed as an individual process in which clients at treatment termination had ceased self-destructive behaviors and acquired skills, leading to changes in self-image and self-acceptance and enabling the creation of a stable and viable way of life. Therapists perceived recovery and a life worth living as mutually reinforcing processes.

**Conclusions:**

The results emphasize early integration of clients’ goals and values in DBT and clarify recovery at treatment termination as a clinically sufficient foundation for a life worth living, contributing to a practice-informed operationalization of recovery within DBT.

**Clinical trial number:**

Not applicable.

**Supplementary Information:**

The online version contains supplementary material available at https://doi.org/10.1186/s40479-026-00352-4.

## Background

Borderline personality disorder (BPD) was long considered a chronic condition with limited prospects for recovery (swe. *återhämtning*) [1, 2]. However, longitudinal follow-up studies suggest that the majority of individuals with BPD no longer meet diagnostic criteria over time, indicating symptom remission over the longitudinal course of the disorder [1, 3]. At the same time, research has shown that symptom remission is not necessarily synonymous with ideal recovery in terms of improved psychosocial functioning and quality of life [1, 3, 4]. This has prompted increasing attention to recovery as a broader construct, encompassing not only symptom change but also clients’ lived experiences of meaningful living [5–7]. Such questions are particularly pertinent in Dialectical Behavior Therapy (DBT), where treatment is explicitly oriented toward helping clients build a life worth living. However, while clients’ perspectives have received increasing attention [5, 7], comparatively little is known about how DBT therapists conceptualize the recovery process [4]. This perspective is clinically relevant, as therapists’ conceptualizations may inform judgments about treatment progress, readiness for treatment termination, and what constitutes clinically meaningful change within DBT practice. Moreover, although existing outcome measures capture central clinical targets in DBT, few explicitly assess how recovery or a life worth living is experienced at treatment termination. Clarifying recovery concepts as articulated in clinical practice may therefore support the development of outcome measures that capture aspects of change that are currently difficult to operationalize and help bridge the gap between measurable treatment targets and broader clinical judgments about recovery.

DBT is currently regarded as the treatment with the strongest empirical support for BPD [8, 9]. Its overarching aim is to reduce problem behaviors and promote more functional problem solving in the client in the collaborative work toward creating “a life worth living” [10], which refers to a life with sufficient positive elements for the individual to want to live [11]. The treatment is phase-specific and organized according to a set of priorities based on a level classification of the client’s problems, which results in a hierarchy of what should be treated and when [8]. Stage 1 is primarily focused on establishing safety and stability by increasing the client’s behavioral control. Reducing life-threatening behaviors is prioritized, followed by therapy-disruptive behaviors and behaviors that impair quality of life [12]. Stage 2 aims to address psychiatric comorbidity, usually post-traumatic stress disorder (PTSD) [12]. The third stage focuses on increasing self-respect and achieving individual goals [10]. Clients’ capacities for self-validation, problem solving, and confidence in their own abilities are strengthened to support basic life satisfaction and self-respect [8]. Stage 4 is dedicated to dissolving feelings of incompleteness and fostering a sense of freedom and sustained life satisfaction [12]. Linehan [10] has emphasized that preparation for treatment termination should begin early and recur throughout therapy, with successful termination requiring the generalization of interpersonal skills beyond the therapeutic context. However, the level of recovery considered sufficient for treatment termination remains undefined in the literature, beyond the broad goal hierarchies specified for each treatment stage [10].

### Recovery

*Recovery* is commonly defined as a restoration or return to health following illness or injury [13]. While some authors conceptualize recovery as an outcome following a recovery process [14], others describe it as an inherently active and ongoing process [15]. The term has been used inconsistently across contexts, with its shifting meanings reflecting broader societal negotiations about the aims and functions of psychiatric care [16]. In the context of BPD, prior research suggests that the concept of recovery may insufficiently capture clients’ subjective experiences. Specifically, the term has been criticized for its binary framing, implying recovery as either achieved or not [5], and for suggesting a return to a prior state, which may be undesirable given the developmental nature of the disorder’s symptoms [17].

There is no consensus regarding what level of recovery should be considered sufficient to warrant treatment termination [16]. Recovery has traditionally been operationalized through quantitative indicators of symptom reduction and psychosocial functioning, commonly referred to as *clinical recovery* [4, 7]. While these indicators capture important aspects of change, they tend to underrepresent clients’ subjective experiences and individual variation in recovery trajectories [4]. In contrast, the concept of *personal recovery*, originating in person-centered qualitative research, foregrounds clients’ lived experiences and conceptualizes recovery as an inherently individual and subjective process [7]. Personal recovery is characterized by shifts in values, skills, attitudes, goals, emotions, and social roles. It encompasses the pursuit of a satisfying, hopeful life despite ongoing limitations related to illness [18]. In the present study, recovery refers to the client’s situation when treatment is judged by the therapist to be ready for termination within a time-bounded outpatient DBT context. In this sense, recovery does not denote an ideal or complete state of psychological health, but rather being recovered enough for discharge. Analytically, it is therefore important to distinguish between (i) ideal recovery, (ii) recovery sufficient for treatment termination, and (iii) termination primarily determined by time or system constraints. The present study focuses on therapists’ descriptions of recovery in the second sense.

Previous research has demonstrated that personal recovery may be experienced despite periodic recurrence of psychiatric symptoms. Against this, ideal recovery from BPD has been conceptualized from a broader perspective that extends beyond symptom remission to include psychosocial dimensions, such as the presence of satisfying relationships and stable identity markers [17]. Turner et al. [7] argue that ideal recovery from BPD encompasses both clinical and personal dimensions, which are best understood as complementary: reductions in life-threatening behaviors and hospitalizations are necessary to sustain social relationships, just as increased hope and self-confidence may mitigate the despair that fuels such behaviors.

Qualitative studies examining clients’ perspectives have identified several components that may characterize ideal recovery from BPD. Consistent with clinical recovery frameworks, these include reductions in life-threatening and self-destructive behaviors, as well as decreased need for inpatient admissions [5, 7]. Improvements in disorder-specific symptomatology, such as impulsivity-related risk-taking and disordered eating have been identified, alongside reductions in comorbid psychiatric symptoms [7]. Moreover, the acquisition and flexible use of DBT-specific skills has been associated with enduring gains in behavioral flexibility and emotion regulation capacity [5, 7, 19, 20], enabling individuals to respond adaptively rather than impulsively across both every day and high‐stress situations. This expanded skill repertoire has been shown to foster a greater sense of agency, self-respect, and confidence in navigating interpersonal and intrapersonal challenges [19]. Ideal recovery has further been described as involving positive transformations in self-perception, identity, and life outlook, with a more affirmative self-image marked by increased self-knowledge and self-acceptance as central features [5, 7, 20]. Clients have additionally reported experiencing a sense of liberation from the past and renewed hope for the future [7, 20]. Satisfactory social relationships represent another recurrent aspect of ideal recovery [5, 7, 19, 20]. Research in this area has highlighted clients’ use of relationship skills, including a strengthened capacity to set and maintain appropriate personal boundaries [7] and to assert one’s needs effectively [20]. Reduced social isolation, greater tolerance of fear of abandonment, and enhanced courage to show vulnerability in close relationships have likewise been emphasized [5]. Clients have described increased openness toward others, which has been associated with greater closeness and reciprocity in relationships [19]. Finally, previous studies consistently underscore the importance of participation in enriching social contexts and purposeful activities as part of the recovery process [7, 19]. This includes sustained engagement in valued social roles and community involvement, with particular emphasis on stable vocational functioning [5, 20].

### A life worth living

Within DBT, recovery at treatment termination cannot be fully understood without considering its relation to the treatment’s overarching goal of helping clients build a life worth living. Linehan [10] has described psychotherapy as an important step toward a life of acceptable quality, while emphasizing that many of the answers to how such a life develops are to be found beyond therapy. Since its inception, DBT has been framed not merely as a suicide prevention treatment, but as a treatment oriented toward helping clients build a life worth living [10]. Accordingly, its core components include skills to enhance clients’ access to positive experiences, both immediately, through increased engagement in pleasurable activities, and over time, by establishing value-driven long‐term goals [21]. The term ”a life worth living” refers to a life that contains sufficient positive elements to motivate waking up each morning, although it does not imply a life free of pain or negative experiences [11]. However, research exploring recovery-related goals and the operationalization of a life worth living remains scarce, and no consensus definition has been established. This may reflect the inherently idiosyncratic nature of what constitutes a life worth living, which depends on individual life experiences and value systems. However, given that this construct represents DBT’s overarching treatment aim [21], this paucity of conceptual clarity constitutes a knowledge gap warranting further investigation.

Liljedahl et al. [6] found that clients described experiencing a life worth living four to six years after completing DBT, perceiving that the trajectories of recovery and a life worth living coalesced as overlapping yet distinct constructs. This temporal emergence aligns with previous findings indicating that ideal recovery develops over time (e.g. 3). However, little is known about how a life worth living corresponds to recovery at treatment termination, and as far as known, no qualitative research has explored the perspectives of DBT therapists. Examining these could provide insight into the extent to which therapists’ understanding of recovery and a life worth living correspond with clients’ lived experiences. Furthermore, there are currently few well-established criteria or instruments for assessing whether, and to what extent, clients achieve recovery and a life worth living following DBT. Accordingly, more in-depth exploration of these constructs may help inform the development of outcome measures that are valid, reliable, and clinically meaningful.

The aim of this study is to deepen the understanding of how DBT therapists describe recovery sufficient for treatment termination in clients with borderline personality disorder. Furthermore, the study aims to explore how DBT therapists perceive the correspondence between recovery at treatment termination and the concept of “a life worth living.” Accordingly, the study addresses the following research questions: (1) How do DBT therapists describe recovery sufficient for treatment termination in clients with borderline personality disorder? (2) How do DBT therapists relate recovery sufficient for treatment termination to the concept of “a life worth living”?

## Methods

### Sample and recruitment

The participants consisted of eight DBT therapists from four distinct geographical regions in Sweden. At the time of data collection, the majority were practicing within adult psychiatry, while two participants were employed in child and adolescent psychiatry. Several participants, however, had clinical experience providing DBT to both adult and adolescent populations. Participants’ ages ranged from 34 to 60 years (median = 55), comprising two men and six women. Their clinical experience in DBT varied from 2.5 to 23 years (median = 17). Six participants were licensed psychologists, three of whom also held licenses as psychotherapists. One participant was a psychiatric aide with foundational psychotherapy training, and one participant was a licensed psychotherapist. Several participants had completed intensive DBT training and received additional education in DBT Prolonged exposure. All participants adhered to a CBT-oriented therapeutic approach and were asked to reflect specifically on their clinical experience of providing DBT to clients diagnosed with BPD. Although participants occasionally referred to heterogeneity within their clinical caseloads, including clients with comorbid conditions, these reflections were contextualized as part of everyday DBT practice rather than as a shift in diagnostic focus. In the participating services, DBT was delivered in a time-limited outpatient care context, typically as a comprehensive program lasting approximately 12–18 months and comprising individual therapy, skills training, and consultation team meetings.

Participants were recruited via email using the DBT Sweden mailing list in December 2024, with a follow-up reminder sent in January 2025. At the time of recruitment, the mailing list comprised approximately 287 members. Eleven therapists responded, all of whom met the inclusion criteria. Of these, ten responded within a time frame that allowed participation. The inclusion criteria specified completion of formal DBT training and a minimum of one year of practical DBT experience. From the respondents, participants with the longest DBT clinical experience were prioritized for inclusion. The final number of participants was determined based on the concept of data saturation, defined as the stage at which new data no longer yields additional meaningful information [22]. However, given that saturation depends not only on the number of participants but also on analytical depth [23], a minimum target of seven interviews was established. After seven interviews, the sample was assessed as analytically saturated and no additional interviews were conducted.

### Procedure

Between December 2024 and February 2025, interviews were conducted based on a semi-structured interview schedule (see Supplementary material). Prior to the main interviews, a pilot interview was carried out with a DBT therapist from the author’s professional network. The pilot participant was a former colleague, met all inclusion criteria, and the interview was the first to take place during the data collection period. As the pilot interview required only minor adjustments to the interview schedule, it was incorporated into the final dataset and analyzed alongside subsequent interviews. To mitigate potential role or relationship effects, the voluntary nature of participation was emphasized, and the pilot interview was treated analytically in the same manner as the remaining interviews.

Seven interviews were conducted remotely via Zoom, and one took place in person. The digital format facilitated participation from multiple regions across Sweden. All participants received written information about the study prior to participation and provided informed consent before data collection, including consent to audio recording and third-party AI-based transcription of de-identified data. Participants were instructed not to disclose identifiable patient information. Interviews were conducted in Swedish and audio-recorded via Zoom, after which only audio files were retained. The interview duration ranged from 27 to 54 min, with an average of 44 min. Following each interview, demographic information, including gender, age, education, and clinical focus, was collected to support reflexive consideration of how participant characteristics might shape the results. Transcription was carried out using an AI transcription service (Transkiptor) [24]. All audio files were de-identified by the author prior to upload, including removal of names, workplaces, and geographical locations. Files were stored temporarily by the transcription service and deleted upon completion of the transcription process. AI-generated transcripts were checked and corrected against the original audio recordings to ensure accuracy and produce verbatim transcripts. Audio files and transcripts were stored on password-protected devices accessible only to the author and supervisor. Quotations included in the manuscript were translated into English by the author. To preserve semantic accuracy, translations were checked against the Swedish transcripts and original audio recordings. For culturally or clinically loaded terms, dictionaries and contextual comparison were used to identify translations that best captured the intended meaning rather than providing literal equivalents.

### Data analysis

In accordance with Braun and Clarke’s model (2006), reflexive thematic analysis was conducted, a method whose flexibility makes it possible to capture the richness and complexity of qualitative data [25]. This study adopted a realist approach, focusing on participants’ accounts of their understandings of recovery, rather than on language use as a social construction [26]. An inductive approach was employed, in which codes and themes were developed from participants’ accounts in relation to the study aim and research questions, focusing analytic attention on therapists’ conceptualizations of recovery rather than on pre-existing theoretical frameworks. Themes were not pre-defined but developed iteratively through engagement with data extracts, codes, and emerging patterns. Coding began at the semantic level, focusing on explicit meanings and patterns in therapists’ descriptions. Latent coding was subsequently used to explore underlying assumptions, implicit meanings, and patterned ways of understanding recovery across accounts. Such interpretations were generated through close engagement with participants’ descriptions and were used to deepen understanding of patterns identified in the semantic data.

The analysis followed the six phases outlined by Braun and Clarke. Familiarization began during transcription checking and continued through repeated reading of the dataset. The dataset was coded systematically in digital format, with codes organized alongside excerpts from each interview. Codes were grouped into preliminary themes reflecting shared patterns of meaning. Throughout this phase, analytic reflections on theme boundaries and interrelations were documented in a reflexive journal and discussed during supervision. Themes were subsequently reviewed and refined in relation to both individual extracts and the dataset. Definitions were developed for each theme to capture their core meaning and delineate thematic boundaries. Writing was treated as an integral analytic process, and illustrative quotations were selected to support the final thematic structure. In the quotations presented below, ellipses in brackets ([…]) indicate omitted text, and italics denote emphasis added for clarity. An illustrative example of the analytic process is presented in Table 1.

**Table 1:** Example of the analytic process from data extract to theme

Data extract	Code	Subtheme	Theme
“If you haven’t gotten closer to your goals, if you’re still actively self-harming and haven’t really cut back on your self-harming, then this treatment hasn’t been effective in that way.”	Sufficient recovery involves cessation of self-harm	Having left self-destructive behaviors	From suffering to a life of normal freedom

### Reflexivity

To address the inherent subjectivity in qualitative analysis, reflexivity constituted a critical component of the analytical process [22]. A reflexive journal was maintained throughout data collection and analysis to document analytic decisions and potential influences on the author’s positioning. The study was conducted within a Western psychiatric context, where individual and medicalized understandings of mental illness are prominent. Given that concepts of recovery are culturally situated [27], it is possible that this context influenced both participants’ accounts and the analytic process. At the time of the study, the author was in the final term of the Study Programme in Psychology. While not trained as a DBT therapist, the author had received university-level teaching in DBT and had previously observed a DBT skills training group during clinical placement. This positioning facilitated understanding of DBT terminology while also necessitating reflexive attention to avoid reproducing model-consistent assumptions. The author’s clinical orientation is CBT, alongside an understanding of BPD informed by the feminist perspective articulated by Kåver and Nilsonne [28], which emphasizes the role of social norms, gendered power structures, and cultural expectations. This perspective informed analytic sensitivity to how therapists described recovery in relation to autonomy, safety, and self-acceptance, while the reflexive journal was used to constrain over-interpretation beyond participants’ accounts.

## Results

### Recovery sufficient for treatment termination in borderline personality disorder

**Fig. 1:**
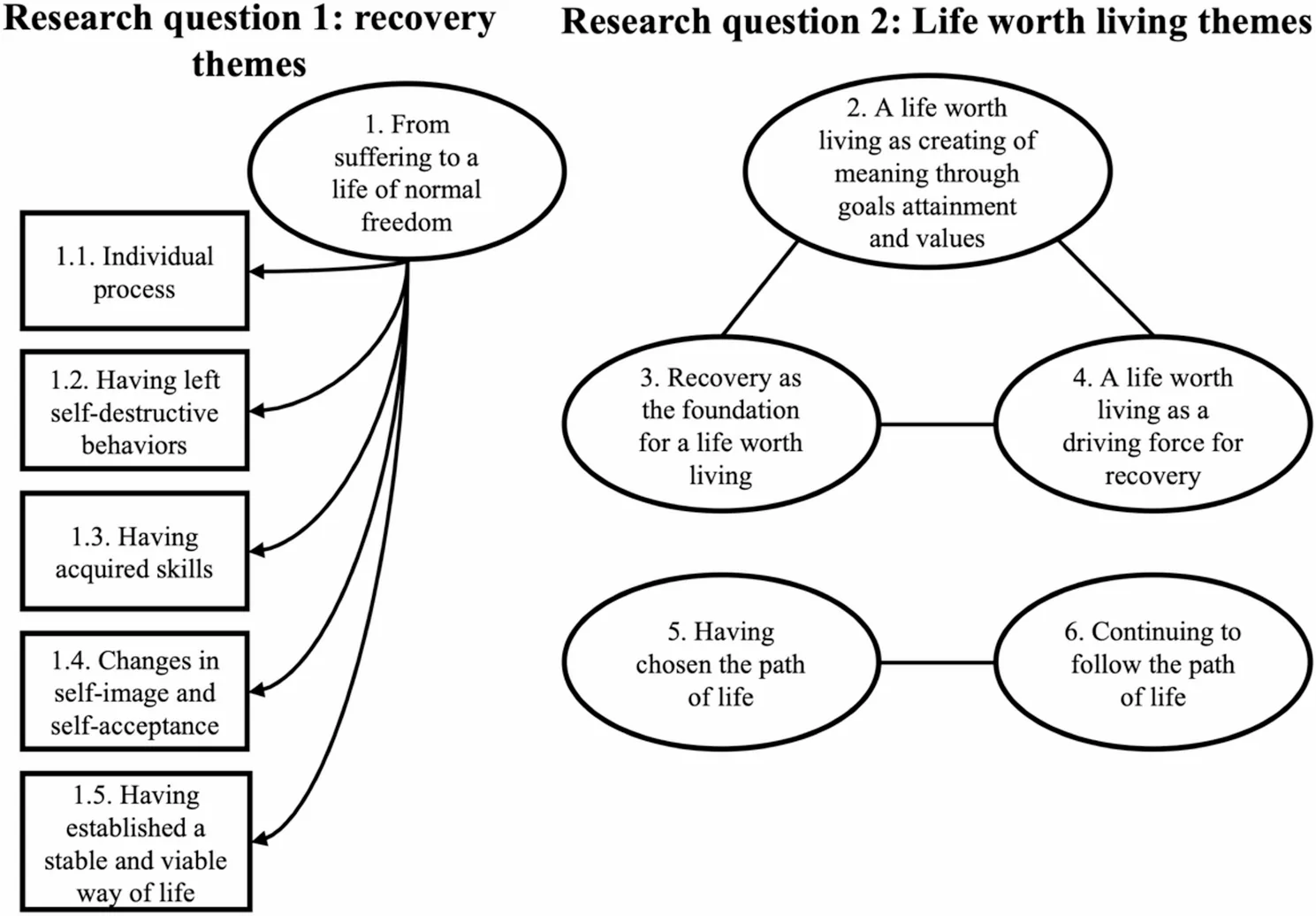
Thematic map of therapists’ conceptualizations of recovery sufficient for treatment termination and a life worth living. *Note* The map illustrates relationships between themes derived from reflexive thematic analysis. Arrows indicate hierarchical relationships between themes and subthemes, whereas connecting lines indicate relationships between themes. The map is intended as an analytic synthesis of the findings rather than a temporal or causal model

The thematic analysis resulted in one main theme and five sub-themes that captured recurring patterns in therapists’ descriptions of recovery at treatment termination (see Fig. 1): (1) From suffering to a life of normal freedom, (1.1) Individual process, (1.2) Having left self-destructive behaviors, (1.3) Having acquired skills, (1.4) Changes in self-image and self-acceptance, and (1.5) Having established a stable and viable way of life.

### Theme 1. From suffering to a life of normal freedom

Across accounts, therapists described recovery sufficient for treatment termination as a transition from overwhelming suffering to a life characterized by what they referred to as normal freedom. Rather than the absence of distress, this freedom denoted liberation from the degree of emotional suffering and behavioral dysregulation that had previously rendered life unmanageable. Prior to treatment, clients’ emotions, cognitions, and behaviors were described as chaotic and marked by emotional dysregulation, with suicidal, impulsive, and other self-destructive behaviors functioning as maladaptive attempts to regulate intense negative affect. By contrast, at treatment termination, clients were described as having developed sufficient emotion regulation capacities to tolerate negative thoughts and feelings while maintaining behavioral flexibility and autonomy in everyday life.

So, recovery could be when you have, when you know how to use your inner compass. That you can be attentive and mindful that now I am reacting, and be able to take care of the feelings and thoughts that are activated in such a way that I can continue to have negative thoughts, negative feelings, and still act in a flexible and… There is this concept of psychological flexibility, and I think that… That’s it, the ability that we work hard to find in some way. (P1)

Therapists emphasized the importance of conveying to clients that experiencing a certain degree of distress constitutes a natural and unavoidable part of life, even after treatment. Consistent with the biosocial model, emotional vulnerability was described as a persistent trait in clients with BPD, implying that even sufficiently recovered clients may continue to experience intense emotions and require ongoing use of emotion regulation strategies. From this perspective, normal freedom reflected the ability to navigate life’s challenges without being dominated by emotional suffering, rather than a return to an idealized state of emotional stability.

#### Individual process

Recovery was conceptualized as an individual process that could manifest differently among clients at treatment completion. The extent of a client’s progress, as well as their specific life circumstances at treatment termination, was described as shaped by personal life goals, psychiatric comorbidities, individual background, and the limitations or opportunities arising from their unique situation.

It can look different for different people. It is highly individual. A goal may not yet have been fully achieved when treatment is coming to an end. The person may not have started studying but may have begun work training and increased their activity level. They may not yet have decided which educational path they want to pursue. But if we have agreed to end treatment after that contract period, then this is something to include in a maintenance plan. (P4)

Therapists described recovery as a dynamic and ongoing process without a definitive endpoint, yet emphasized that treatment itself was temporally limited. Several therapists noted that treatment termination is not necessarily contingent upon the therapist’s assessment of a client’s complete recovery. Rather, termination often depends on available treatment duration and the therapist’s appraisal of the client’s residual care needs at the end of the allotted time. Consequently, several therapists reported seldom employing the concept of “recovery” explicitly in clinical practice, favoring instead situation-specific descriptions such as “progress in treatment,” “finding one’s inner compass,” “feeling better,” or “building skills” to characterize clients’ developmental trajectories.

#### Having left self-destructive behaviors

A prerequisite for recovery sufficient for treatment termination was identified as the cessation of self-harm, life-threatening, and other serious self-destructive behaviors. Therapists emphasized that this remained necessary even when clients had made progress in other areas, such as achieving personal goals.

Within DBT, I see the cessation of dangerous and self-destructive behaviors as essential to recovery. Other aspects may still be evolving, but those behaviors need to have stopped. (P3)

#### Having acquired skills

Therapists identified skill acquisition and flexible skill use as central to recovery at treatment termination, with sufficient skills indicating readiness for treatment termination and the capacity to continue building a desired life independently.

Yes, skills for dealing with really difficult situations, crises, but also everyday situations. Being able to organize yourself, understand what is important and not become too fragmented, overwhelmed, and stressed in situations. So yes. So that you can use these skills in situations where you notice that it’s not like… Those skills are not activated by themselves, naturally. And I need to stop and feel and follow this inner compass. (P1)

#### Changes in self-image and self-acceptance

At treatment termination, recovery was characterized by positive changes in clients’ self-image and self-acceptance. Clients were described as having developed deeper self-awareness, a more consolidated sense of identity, and improved insight into their challenges. The ability to identify remaining life difficulties, recognize the need for further personal work, and clarify personal values in these areas was highlighted as central. Increased acceptance of oneself, including one’s level of functioning and past painful experiences, was considered particularly important. Therapists emphasized the significance of clients’ capacity to confront and radically accept even traumatic experiences.

And when I think about it, this person has recovered. That often means that they have been able to, like, face, accept and cope with the feelings related to what they have been through in their life. Depending on what that may be. (. …) Not only that you have learned these DBT skills, but also that you can deal with what has been in your life, the painful points in your life. Yes, which can be… It could be anything from sexual abuse throughout childhood to more, yes, more or less traumatic events or traumatic invalidation (…). (P3)

#### Having established a stable and viable way of life

Clients sufficiently recovered at treatment termination were depicted as having established a stable and viable way of life. Therapists emphasized the importance of a secure everyday environment, free from exposure to harm or threat, and the establishment of stable routines, alongside engagement in activities that anchored daily life and supported continued functioning beyond treatment.

Yes, I have [at treatment termination] what is important. It’s my animals. What’s important are my relationships. What’s important is my involvement in (…) the World Wildlife Fund or whatever it is? Yes, yes, something that kind of anchors me in life. (P1)

Such engagement could take diverse forms depending on individual circumstances and included vocational involvement adapted to personal capacities and access to supportive relationships. Strengthening clients’ social networks could involve reconnecting with family members or establishing new relationships based on mutuality and respect. In this sense, recovery at treatment termination was understood as having created the conditions for personal growth beyond treatment, rather than as an endpoint defined by fulfillment.

### Recovery sufficient for treatment termination and a life worth living

Across accounts, the thematic analysis resulted in five main themes reflecting how DBT therapists relate recovery sufficient for treatment termination to the concept of a life worth living (see Fig. 1): (2) A life worth living as creation of meaning through goal attainment and values (3), Recovery as the foundation for a life worth living (4), A life worth living as a driving force for recovery (5), Having chosen the path of life, and (6) Continuing to follow the path of life.

### Theme 2. A life worth living as creation of meaning through goal attainment and values

The results showed that a life worth living was defined as a subjective experience of meaning characterized by the attainment of foundational goals and regular engagement with personal values. The concept was described in opposition to self-destructive behaviors and suicidality; therapists described an active choice to engage in living, rather than persistently wishing to die, as a central marker of a life worth living. Therapists emphasized that this required a subjective sense of hope for the future, along with a perception that life holds intrinsic meaning and value, even during challenging periods. The attainment of foundational goals was highlighted as a central aspect, including basic security, at least one stable relationship, and some engagement in activities that provide a sense of satisfaction, alongside individually determined goals. While therapists’ descriptions of a life worth living in this sense overlapped with elements present at treatment termination, they emphasized that a life worth living remained fundamentally a subjective orientation toward meaning.

But I think that *both*, like, valued direction, it’s a bit existential. (. …) But at the same time, there is this more standard CBT part in that you really have to formulate concrete goals. The concrete goals are what will also make life worth living, what will create meaning and a sense of competence. (P4)

In addition to supporting clients’ pursuit of concrete, achievable goals, the work of cultivating a life worth living was described as closely related to values work. In this sense, therapists emphasized the importance of exploring and clarifying personally meaningful values that could counter self-destructive tendencies and deepen the experience of meaning, while also translating these values into concrete, goal-oriented therapeutic work.

### Theme 3. Recovery as the foundation for a life worth living

The analysis revealed that a certain degree of recovery was viewed as an essential precondition for achieving a life worth living. Since self-destructiveness was considered incompatible with the concept, it was emphasized that it was impossible for a client to achieve a life worth living without having recovered to some extent. Additionally, achieving a life worth living was considered unattainable during DBT stage 1, as therapists noted that self-destructive behaviors must first be reduced significantly. Some therapists included the beginning of DBT stage 2, arguing that decreased emotional avoidance was also essential. Thus, the development of a life worth living was described as emerging within the recovery process and continuing to evolve over time.

First, you need to be able to validate yourself. (…) Instead of only criticizing and judging yourself, to change that to self-validation and start taking care of yourself. And only then will there be plenty of room to think about what I want, who I am and what would be good for me. Like… Allowing yourself to search. (P5)

Therapists emphasized that gradual reductions in psychiatric suffering over the course of recovery directly contributed to a life worth living by enabling clients to move from self-criticism toward self-validation and self-care, and thereby to begin exploring what they wanted from life and what might make it worth living. As suffering and suicidal ideation decreased, clients were described as becoming more able to engage in activities that fostered positive affect over time, supporting a sense that life was increasingly worth living.

### Theme 4. A life worth living as a driving force for recovery

The analysis indicated that a life worth living functioned as a central motivational orientation within the recovery process, providing direction and sustaining engagement throughout treatment. A life worth living was described as underpinning therapeutic work by motivating clients to persist with skills use and to tolerate short-term distress instead of resorting to self-destructive behaviors. Early engagement with the concept of a life worth living was therefore described as important, as it supported the identification of personal goals and values and helped disrupt ongoing cycles of self-destructive behavior. In the absence of such values-oriented work, therapists described maladaptive patterns as more likely to persist.

Yes, but if they don’t find themselves and what they want, how they want to live, then they will live as they did [before treatment]. Then this woman who is treated very badly by her husband will continue to live with him. Yes, then she will continue to have her severe anorexia and self-harming behavior, probably, she will not feel better. So, you have to find yourself, because when I feel that I deserve not to be abused every day, I will move into my own apartment. (P5)

### Theme 5. Having chosen the path of life

Across narratives, therapists described that clients who were sufficiently recovered at treatment termination had chosen the path of life, suggesting they had moved toward, or attained, a life worth living by the end of treatment. This was described as characteristic of most clients considered sufficiently recovered, although not universally achieved. Even clients who had not yet realized their life goals or consistently experienced life as worth living were depicted as having chosen engagement with life over self-destructive alternatives.

A patient might say, now I have a life worth living. (. …) It’s a bit like choosing, you’ve made a choice, so to speak. That I am now on this path, where I… Where I devote myself to being active in my life and building that life and doing the things I want or need, which is the life that is worth living. So yes, the patient can say that life, because it is a life worth living today, I don’t think, I no longer think that I should take my own life. I think that I should live. (P3)

### Theme 6. Continuing to follow the path of life

Therapists further highlighted that clients continue shaping and redefining a life worth living after treatment completion, as their needs and aspirations naturally evolve. Several therapists described DBT’s fourth phase as dedicated to living consciously in line with one’s values while continuing to utilize skills. Thus, the recovery process and a life worth living were understood as closely intertwined, extending beyond the formal conclusion of treatment.

We do evaluations after the end of treatment, and they report that… That they are in their lives and that they really… Really see it as worthwhile to continue on that path. I think life is a journey, and it is for everyone. And who says when you have reached your goal, and you know, what we want in our lives for it to be worth living also changes over time. But I think they really have the opportunity to revise it, make other decisions. Make sure they get what they think they want, when it changes. (P6)

## Discussion

The present study aimed to deepen the understanding of how DBT therapists describe recovery sufficient for treatment termination in clients with BPD, and how this form of recovery relates to a life worth living. The results of the analysis show that DBT therapists described recovery sufficient for treatment termination as a transition from suffering toward a life of normal freedom. This transition was conceptualized as an individual process in which clients had ceased self-destructive behaviors and acquired skills, leading to changes in self-image and self-acceptance and enabling the establishment of a stable and viable way of life. These descriptions overlap with client-centered qualitative accounts of recovery from BPD as an individualized process involving improvements in both psychosocial functioning and internal psychological processes [5, 7, 19, 20], enabling a fulfilling life despite remaining challenges [18, 29]. More broadly, they also resonate with recovery literature that conceptualizes recovery as extending beyond symptom reduction to include agency, self-acceptance, meaning, and participation in everyday life despite ongoing vulnerability [15, 18].

Therapists described recovery sufficient for treatment termination as providing the foundation for a life worth living, while a life worth living was framed as a driving force within the recovery process. These results align with theoretical proposals suggesting that recovery and a life worth living should not be treated as identical constructs, and that a certain degree of recovery is required to experience a life worth living [6]. In the present study, recovery at treatment termination was not conceptualized as meaning or subjective fulfillment. Instead, recovery was understood as the establishment of sufficient emotional, behavioral, and contextual stability to enable ongoing participation in everyday life. From this perspective, recovery creates the conditions under which a life worth living may gradually develop, but does not in itself require the experience of meaning. This distinction is consistent with DBT’s emphasis on helping clients build stable lives, while understanding a life worth living as something that must remain client-defined [10]. Thus, recovery at treatment termination was not understood as an ideal or complete state of recovery, but as a clinically sufficient level of change that allowed treatment to be concluded.

### Recovery sufficient for treatment termination

Therapists’ descriptions of recovery sufficient for treatment termination align with the definition reported by Comtois et al. [29], in which recovery was defined as an individual process that enables the client to engage in a satisfying and active societal life despite any remaining challenges. In the present study, therapists’ accounts did not explicitly differentiate between clinical and personal recovery as described by Slade et al. [16]. Instead, their descriptions included components related to clinical recovery, such as reduced self-destructiveness and improved psychosocial functioning, and aspects associated with personal recovery, such as increased self-awareness, self-acceptance, and hope for the future. This is consistent with Turner et al.‘s [7] characterization of recovery from BPD as a process in which clinical and personal dimensions should be considered complementary. In this respect, the findings are also consistent with McCusker et al. [17], who argue that meaningful change in BPD cannot be adequately understood through symptom-focused measures alone.

Therapists conceptualized recovery at treatment termination as a transition from suffering to a life of normal freedom. In this context, freedom did not refer to the absence of emotional pain, but to greater freedom from being controlled by extreme emotional states and self-destructive behaviors. It denoted a greater capacity to tolerate distress, act flexibly in everyday life, and move toward longer-term goals. Freedom has long been recognized as an explicit target and potential outcome of DBT skills training, particularly within the mindfulness, emotion-regulation, and distress‐tolerance modules [12]. However, the present results suggest that therapists regarded such freedom not merely as a distal product of skills acquisition, but as a central experiential marker of recovery at treatment termination. In this respect, the results align with Linehan’s [10] biosocial model, as therapists linked recovery to a reduced degree of emotional dysregulation and greater behavioral flexibility. This also adds specificity to broader discussions of recovery in BPD, as Ng et al. [4] note that recovery is understood differently across consumer, clinician, family, and carer perspectives. More specifically, the present results suggest that therapists emphasized subjective freedom from overwhelming emotional suffering as a salient aspect of recovery at treatment termination. This may represent an important, but relatively underexamined, dimension of recovery.

Results further support conceptualizations of recovery as a dynamic and non-linear process, consistent with prior accounts emphasizing fluctuation and setbacks in recovery from BPD [5, 17, 20]. Therapists highlighted reductions in self-destructive behaviors and the acquisition of DBT skills as central to recovery, aligning with previous research identifying skills training as a core mechanism of change [5–7]. Extending this literature, the present results suggest that therapists viewed the attainment of a sufficient skill repertoire not only as supportive of long-term recovery, but also as a practical indicator of readiness for treatment termination, an aspect not explicitly addressed in earlier reviews [4]. Beyond behavioral and skill-based change, therapists emphasized shifts in self-image and self-acceptance, as well as the establishment of a stable and viable everyday life, as integral to recovery at treatment termination. These elements overlap with previously reported indicators in client-centered studies [4, 7, 19, 20], while reflecting therapists’ focus on functional stability rather than subjective fulfillment at the point of discharge. Together, these findings contribute to a more nuanced understanding of recovery at treatment termination and may inform the development of outcome measures that capture clinically meaningful change without conflating recovery with the experience of a life worth living. However, these findings do not justify treating therapists’ descriptions as definitive criteria for recovery, nor do they support conclusions about clients’ subjective experiences or long-term outcomes following treatment termination.

Therapists reported infrequent use of the recovery term in clinical practice. One possible explanation is that, in the absence of shared and operationalized anchors for what constitutes recovery sufficient for treatment termination, the term may have limited utility in routine treatment evaluation [16, 17]. Avoiding a singular recovery label may reflect an effort to reduce pathologizing or stigmatizing framings, thereby supporting more nuanced and person-centered goal formulation [16]. Nevertheless, therapists described actively attending to outcomes congruent with their own descriptions of recovery, suggesting that these changes were considered clinically salient even when not summarized under a common label. This lack of shared terminology may therefore risk ambiguity in how “successful treatment termination” is communicated and evaluated, beyond the overarching DBT aim of supporting a life worth living. Taken together, these results suggest a possible difficulty in evaluating individualized, termination-relevant change when common terminology is not routinely used.

### Recovery at treatment termination in relation to a life worth living

Therapists conceptualized a life worth living as a subjective experience of meaning grounded in both foundational goal attainment and ongoing engagement with personal values. Consistent with Linehan’s framework [11], access to safety, relative self-sufficiency, and social connectedness were described as necessary conditions for clients to begin experiencing life as worth living. In this study, such goal attainment did not constitute recovery per se, but rather contributed to the emergence of a life worth living once a sufficient level of recovery had been achieved. Living in regular contact with personal values was also identified as a central component of a life worth living. While values-based elements are embedded within DBT skills training, most notably in modules focused on accumulating positive experiences over time [12], therapists emphasized values clarification as a broader orienting process that gives direction to therapeutic work. In this sense, the present results extend existing DBT theory by articulating how goal attainment and values engagement jointly contribute to clients’ subjective experience of meaning within treatment contexts.

Therapists distinguished between recovery sufficient for treatment termination and a life worth living, describing them as conceptually distinct but clinically related phenomena. This distinction aligns with service-user accounts reported by Liljedahl et al. [6], in which recovery and a life worth living were identified as related yet separable experiences. Seen in relation to wider recovery literature, therapists’ descriptions suggest that a life worth living shares certain themes with broader recovery-related themes of meaning and valued action [15, 18]. Recovery at treatment termination, however, was not conceptualized as the attainment of meaning or subjective fulfillment, but as the establishment of sufficient emotional, behavioral, and contextual stability to allow clients to participate in everyday life without reliance on self-destructive behaviors. From this perspective, recovery creates the conditions under which a life worth living may be developed, while remaining conceptually distinct from it. Recognizing that these constructs are not interchangeable has important clinical implications, given Linehan’s [10] definition of a life worth living as DBT’s ultimate treatment goal. The results, however, clarify that experiencing a life worth living does not necessarily equate to sufficient recovery from psychiatric pathology and that the need for ongoing treatment may therefore persist.

At the same time, therapists described recovery and a life worth living as dynamically interrelated in clinical practice. A certain degree of recovery, particularly reductions in self-destructive behavior and improved emotion regulation, was viewed as a prerequisite for engaging in valued living. This pattern is consistent with findings reported by Liljedahl et al. [6], in which participants described achieving recovery following symptom remission but prior to the emergence of a life worth living. Conversely, contact with a life worth living was described as a motivating orientation that sustained engagement in recovery work, especially during periods of distress. In this respect, the results suggest that a life worth living functioned not merely as a distal outcome, but as an orienting framework within the recovery process, consistent with broader recovery literature describing recovery as guided by personally meaningful direction rather than symptom change alone [18, 27]. Rather than representing overlapping constructs, recovery and a life worth living were thus described as functionally distinct processes that reciprocally influence one another within and beyond treatment.

Therapists further articulated that, by the end of treatment, many clients deemed sufficiently recovered had already moved toward, or attained, a life worth living, described by therapists as having chosen “the path of life.” This contrasts with findings from Liljedahl et al. [6], where participants described the experience of a life worth living as emerging several years after treatment. Rather than indicating inconsistency, this difference may reflect the distinct perspectives of clinicians and service users, as well as differences between recovery thresholds used in clinical decision-making and clients’ longer-term experiential accounts. These results thus highlight how thresholds for sufficient recovery in clinical contexts may diverge from clients’ retrospective accounts of when a life worth living is fully experienced.

The study’s final theme underscores that completion of DBT does not mark an endpoint of recovery, but rather establishes a foundation for continued self-development [19], and for ongoing construction and revision of a life that is experienced as worth living. In this sense, DBT was described as a treatment that equips clients with the skills and resources needed to sustain recovery processes and to shape a life worth living beyond treatment.

### Methodological integrity

*Methodological integrity* has been proposed by Levitt et al. [30] as a benchmark for credibility in qualitative inquiry, grounded in two core principles: utility and fidelity to the subject. *Utility* concerns the extent to which chosen methods meaningfully address the research questions [30]. In this study, semi-structured interviews were appropriate for eliciting therapists’ conceptualizations of recovery, as the interview guide provided structure, while allowing flexibility for follow-up questions and in-depth exploration. The resulting data corpus exhibited internal coherence, with apparent contradictions deliberately examined to yield a rich, nuanced understanding of recovery as conceptualized by therapists across both adult and child and adolescent psychiatric settings. This thorough grounding of results in the data enhances their *transferability*, understood as their potential applicability to similar clinical contexts [22]. To support readers’ assessment of transferability, contextual information regarding participants, procedures, and service settings was described, although details about specific workplaces and educational backgrounds were omitted to protect participant anonymity, which may limit the evaluation of contextual specificity.

The second principle, *fidelity to the subject*, concerns the researcher’s ability to remain closely attuned to participants’ experiences throughout data collection and analysis [30]. The researcher’s familiarity with DBT terminology and clinical discourse facilitated nuanced probing during interviews, but also required deliberate reflexivity to avoid reproducing model-consistent assumptions. Recruitment through the DBT-Sweden network likely contributed to a high level of model adherence among participants, which may have shaped how recovery and treatment outcomes were articulated. Accordingly, the findings should be understood as reflecting therapists’ perspectives within established DBT practice, and alternative conceptualizations may have emerged in other clinical or organizational contexts. Consistent with a reflexive thematic analytic approach, formal participant involvement in reviewing or validating the findings was not undertaken. Instead, theme development was discussed in ongoing supervision, where critical feedback was provided on the coherence and boundaries of the themes. Analytic credibility was further supported through iterative reflexive engagement with the data, including repeated readings, memo-writing, and critical examination of similarities and divergences across accounts. Throughout theme development, interpretive decisions were grounded in participants’ own formulations, with transparent analytic procedures intended to enable readers to assess the transferability of the results.

### Future research

The results of the present study suggest that the Swedish term for recovery (*återhämtning*) is infrequently used in a systematic manner in clinical practice, highlighting a need for further research on how recovery-related change is conceptualized and communicated within DBT contexts. Future studies could examine whether the development of a more precise or practice-informed terminology might facilitate clearer articulation and evaluation of treatment-relevant change at termination. Importantly, such conceptual work should be grounded in clinical practice and preserve the person-centered orientation identified in this study, including attention to clients’ individual goals, values, and subjective experiences of meaningful change. Empirical efforts to refine or test alternative formulations of the Swedish concept of recovery may contribute to improved operationalization of outcomes that are clinically salient yet currently difficult to capture using existing measures. In the longer term, this line of research may also support the development of a shared language for communicating that recovery from BPD is possible.

### Conclusions and clinical implications

The present study contributes a practice-based conceptualization of recovery sufficient for treatment termination in DBT for BPD. Rather than framing recovery as the attainment of subjective fulfillment or complete symptom remission, therapists described recovery at treatment termination as a clinically sufficient transition from overwhelming suffering toward a life characterized by freedom, stability, and functional autonomy. This transition was marked by the cessation of self-destructive behaviors, the acquisition and flexible use of skills, shifts in self-image and self-acceptance, and the establishment of a stable and viable everyday life that could be sustained beyond treatment. From a clinical perspective, these results suggest that recovery at treatment termination is evaluated not solely through symptom-based criteria, but through a broader appraisal of clients’ capacity to manage distress, maintain safety, and function adaptively in everyday life. The identified dimensions may therefore provide a clinically meaningful framework for structuring termination assessments, supporting shared decision-making about discharge readiness, and clarifying treatment goals for both therapists and clients.

Moreover, the study clarifies that recovery at treatment termination and a life worth living are neither interchangeable nor sequential endpoints. Recovery was conceptualized as creating the conditions under which a life worth living may emerge, while ongoing contact with a life worth living was described as motivating and sustaining engagement in recovery processes. Clinically, this distinction allows therapists to acknowledge meaningful progress and justify treatment termination without requiring clients to report enduring fulfillment or complete resolution of difficulties, while still maintaining a therapeutic focus on values and on exploring what constitutes a life worth living, thereby harnessing the concept’s motivational potential beyond treatment. In this context, the theme of “having chosen the path of life” illuminates how termination readiness in DBT may be understood as an observable shift in orientation rather than as the achievement of an idealized outcome.

It remains unknown to what extent clients who complete DBT and are assessed as sufficiently recovered experience their lives as worth living. However, the results of the present study indicate that DBT therapists commonly perceive this to be the case at treatment termination, while simultaneously emphasizing that recovery does not conclude the work of living. Rather, therapists described treatment completion as marking the beginning of an ongoing process in which clients continue to choose engagement with life and to actively construct, maintain, and revise a life experienced as worth living, drawing on the skills acquired during therapy. In the therapists’ accounts, DBT was understood as enabling clients to move beyond the pervasive suffering that had shaped life before treatment and toward a degree of freedom that makes valued living possible.

## Supplementary Information

Below is the link to the electronic supplementary material.

Supplementary Material 1

## Data Availability

The pseudonymized datasets are not publicly available due to risk of re-identification. Excerpts may be obtained from the corresponding author on reasonable request.

## Declarations

### Supplementary materials

The full Swedish online version, including supplementary materials, is available in the Stockholm University DiVA repository (URN: urn: nbn: se: su: diva-243427).

## Abbreviations

BPD: Borderline personality disorder
DBT: Dialectical Behavior Therapy
PTSD: Post-Traumatic Stress Disorder
