# Supplementary material for: Recovery from borderline personality disorder in relation to the concept of “a life worth living”: a therapist perspective
Source: Borderline Personal Disord Emot Dysregul. 2026 Sep 4;13:27. doi: 10.1186/s40479-026-00352-4 (PMC13570502; doi:10.1186/s40479-026-00352-4)
Supplement: Supplementary file 1 [file 40479_2026_352_MOESM1_ESM.pdf]

Appendix A. Interview guide Summary

Interview focus and structure

The semi-structured interviews were conducted in Swedish and aimed to explore DBT therapists' conceptualizations of recovery at treatment termination, as well as how recovery was understood in relation to the DBT goal of *a life worth living*. To enhance transparency while preserving linguistic and conceptual fidelity, this appendix presents a translated summary of the core interview questions and illustrative probes, rather than a verbatim translation of the original interview guide. The complete interview guide is available in Swedish through the Stockholm University DiVA repository (URN: urn:nbn:se:su:diva-243427).

The interview guide was organized into three main sections, with open-ended questions and flexible follow-up probes used to elicit concrete clinical examples and elaborations.

Section 1: Conceptualizing recovery at treatment termination

- How do you understand *recovery* in clients with borderline personality disorder within DBT?
- What characterizes clients you consider sufficiently recovered for treatment termination?

Section 2: Identifying recovery in clinical practice

- How does recovery manifest during the course of DBT treatment?
- Can you describe a clinical situation in which you judged a client to be sufficiently recovered?

Section 3: A life worth living and recovery

- How do you understand the concept of *a life worth living* based on your clinical experience?
- How do you view the relationship between recovery at treatment termination and a life worth living?

Closing question

- Finally, is there anything else you would like to add or reflect on before we conclude the interview?

Illustrative probes:

- Can you give a concrete example from your clinical experience?
- How did you reason in that situation?
- Can you compare this to a client who was not sufficiently recovered?
- Can you say more about that?
